# Modelling coffee leaf rust risk in Colombia with climate reanalysis data

**DOI:** 10.1098/rstb.2015.0458

**Published:** 2016-12-05

**Authors:** Daniel P. Bebber, Ángela Delgado Castillo, Sarah J. Gurr

**Affiliations:** 1 Department of Biosciences, University of Exeter, Stocker Road, Exeter EX4 4QD, UK; 2 Rothamsted Research, North Wyke, Okehampton EX20 2SB, UK

**Keywords:** climate change, food security, coffee leaf rust, climate reanalysis, plant pathology, epidemiology

## Abstract

Many fungal plant diseases are strongly controlled by weather, and global climate change is thus likely to have affected fungal pathogen distributions and impacts. Modelling the response of plant diseases to climate change is hampered by the difficulty of estimating pathogen-relevant microclimatic variables from standard meteorological data. The availability of increasingly sophisticated high-resolution climate reanalyses may help overcome this challenge. We illustrate the use of climate reanalyses by testing the hypothesis that climate change increased the likelihood of the 2008–2011 outbreak of Coffee Leaf Rust (CLR, *Hemileia vastatrix*) in Colombia. We develop a model of germination and infection risk, and drive this model using estimates of leaf wetness duration and canopy temperature from the Japanese 55-Year Reanalysis (JRA-55). We model germination and infection as Weibull functions with different temperature optima, based upon existing experimental data. We find no evidence for an overall trend in disease risk in coffee-growing regions of Colombia from 1990 to 2015, therefore, we reject the climate change hypothesis. There was a significant elevation in predicted CLR infection risk from 2008 to 2011 compared with other years. JRA-55 data suggest a decrease in canopy surface water after 2008, which may have helped terminate the outbreak. The spatial resolution and accuracy of climate reanalyses are continually improving, increasing their utility for biological modelling. Confronting disease models with data requires not only accurate climate data, but also disease observations at high spatio-temporal resolution. Investment in monitoring, storage and accessibility of plant disease observation data are needed to match the quality of the climate data now available.

This article is part of the themed issue ‘Tackling emerging fungal threats to animal health, food security and ecosystem resilience’.

## 1. Introduction

Fungal pathogens are the most damaging disease agents in global crop production. Despite chemical controls and plant resistance breeding, around one-quarter of global production is lost, enough to feed hundreds of millions [1]. Despite efforts to restrict the spread of diseases via quarantine and other biosecurity controls, fungal pathogens are spreading rapidly to reach their plant hosts around the world [2]. Recent examples of range expansions include arrival of wheat blast (*Magnaporthe oryzae*) into Bangladesh from Latin America [3], the virulent Ug99 race of wheat stem rust (*Puccinia graminis* f. sp. *tritici*) into Egypt from sub-Saharan Africa [4], and orange rust of sugar cane (*Puccinia kuehnii*) into Argentina, probably from Brazil [5].

The impact of resident pathogens varies dramatically in time and space, depending upon factors such as susceptibility of the host crop, evolution of pathogen virulence, disease management strategies and prevailing environmental conditions [6]. The life cycles of many fungal pathogens are strongly determined by weather. Dispersal can be assisted by wind and rain, while germination and infection rates are often dependent upon liquid water on the plant surface (sometimes high relative humidity) and species-specific optimal temperature ranges [7]. The role of weather in determining the likelihood of disease outbreaks is well known to farmers, and numerous risk-forecasting models have been developed to assist in timing management interventions, such as fungicide sprays, for maximum efficiency [8]. In the UK, for example, agronomists predict that the record-breaking warm winter of 2015–2016 will trigger severe outbreaks of several fungal pathogens [9]. The importance of weather has also motivated the application of species distribution models, which employ estimates of species' climatic tolerances, to map potential future geographical ranges of fungal pathogens [10].

Average global surface temperatures have increased by nearly 0.9°C in the century to 2015 [11], accompanied by significant changes in the dynamics of the oceans and atmosphere [12]. Wild populations of plants and animals have shifted their geographical ranges and phenologies in response [13], and changes in crop–pathogen interactions mediated by altered climates are thus a reasonable expectation [14,15]. Attribution of plant disease outbreaks and invasions to particular causes has important policy implications [6], influencing the relative investment in measures such as quarantine, predictive risk models and plant protection—the response to finding that an outbreak was due to the breakdown of plant resistance would differ from that due to weather.

There are two common approaches to investigating the influence of climate change on crop–pathogen interactions. The first, prospective, approach is to compare the predictions of weather-dependent disease risk models driven with current climates and future climate projections, thereby determining how disease impacts might change in future. The risk models vary from mechanistic descriptions of the pathogen life cycle driven by hourly estimates of weather conditions [16,17], to statistical relationships with weather [18] or annual climatic averages [10]. A disadvantage of these projections is that validations of such long-range projections are rare, because they occur in the future (but see [15]). The second, retrospective, approach is to statistically analyse historical patterns and trends in disease distributions or impacts, either for particular case studies [19], or multiple species [20], correlating these with climate trends to determine whether climate change that has already occurred could be responsible for altered disease risk. This mirrors the analysis of wild population responses to historical climate change [13]. While there is some evidence for a response in plant pathogen impacts and distributions to historical climate change [15,20], the method is hampered by missing observational data and the strong influence of other confounding factors affecting plant disease [19,21].

**Figure 1.**
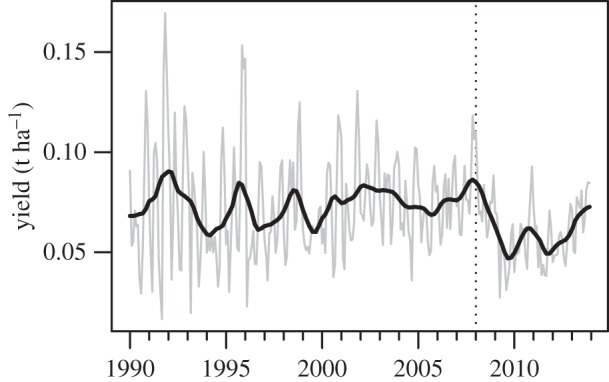
Monthly coffee yield (t ha^−1^) in Colombia, January 1990 to December 2013. Monthly production data [25] were divided by estimates of monthly harvested area derived by six-month moving average interpolation of annual harvested area [26]. Grey line shows monthly yield; black line shows trend derived from seasonal-trend decomposition by loess [27]; vertical dotted line marks January 2008.

Here we propose that combining mechanistic disease models with increasingly sophisticated climate reanalyses [22] will facilitate retrospective modelling of plant disease risk. We illustrate this idea with an analysis of the recent outbreak of coffee leaf rust (CLR), caused by the Basidiomycete fungus *Hemileia vastatrix*, in Latin America [23]. We focus particularly on Colombia, one of the world's largest coffee producers with around one million hectares under cultivation, primarily of the high-value *Coffea arabica* species. CLR is endemic to the centre of origin of coffee in Ethiopia, but has spread to all coffee-growing regions, reaching Brazil by 1970, and Colombia by 1983 [24]. Since then, CLR damage has varied among countries and from year to year. Mean annual production in Colombia is around 60 000 tonnes, which declined by around 40% from 2008 to 2011, increasing again thereafter (figure 1). This production decline has been linked to a severe CLR outbreak that occurred across Colombia and neighbouring Latin American countries from 2008 to 2013 [23]. Several hypotheses have been proposed to explain the recent CLR outbreak, including the evolution of a new, virulent race of the pathogen, changes in plantation management regimes promoting disease development and favourable weather conditions due to climate change [23]. A recent analysis employed a simple correlative approach to test the climate change hypothesis, which indicated that changes in diurnal temperature ranges may have been influential [23]. We develop a mechanistic model of CLR germination and infection risk, and drive this model with the JRA-55 climate reanalysis dataset [28]. We compare the outputs of the risk model with the observed outbreak in Colombia, and test the hypothesis that climate-driven risk has changed significantly over recent decades.

## 2. Material and methods

### (a) Study system

**Figure 2.**
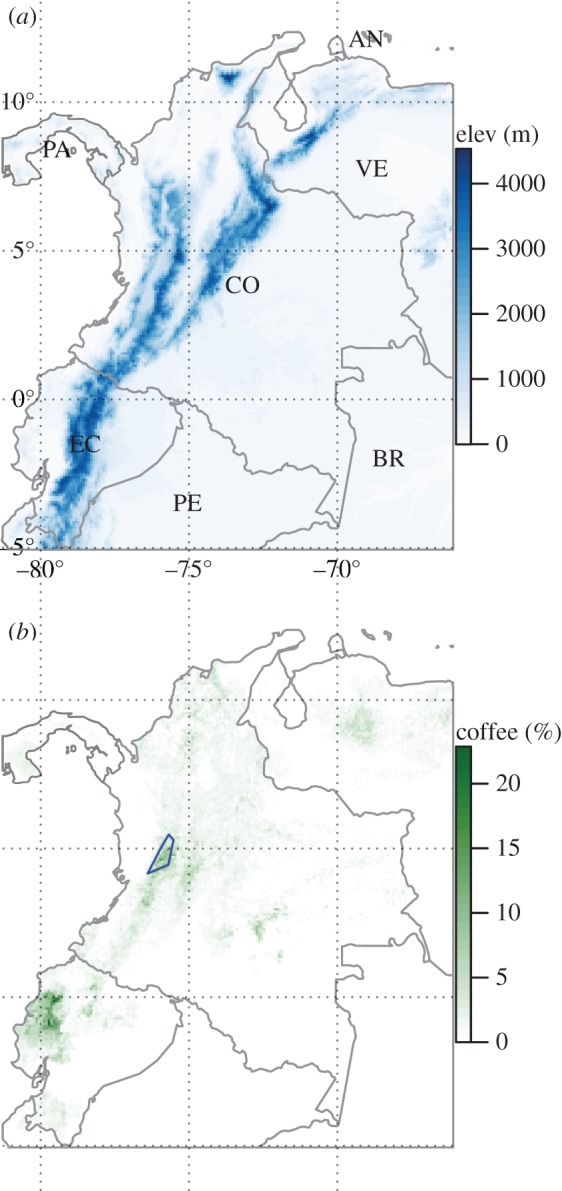
Topography and coffee production. (*a*) Median elevation (m.a.s.l.) from NASA SRTM digital elevation model at 5 arcmin resolution. Labelled countries are Brazil (BR), Colombia (CO), Dutch Antilles (AN), Ecuador (EC), Panama (PA), Peru (PE) and Venezuela (VE). (*b*) Coffee cultivation, percentage of area planted in 2000 at 5 arcmin resolution [29]. The blue polygon encompasses the six UNESCO World Heritage Site areas within the Coffee Cultural Landscape of Colombia.

Our region of interest (ROI) includes Colombia and neighbouring countries (figure 2*a*). Colombia is topographically complex, rising from the Amazon Basin in the southeast to the Andes in the northwest. Temperatures decline with altitude but are largely aseasonal. The majority of the country is classified as Equatorial climate, with a region of Temperate Oceanic climate on the Venezuelan border (Köppen–Geiger classification). Coffee-producing areas were identified using estimates of crop distributions in the year 2000 [29]. Coffee harvested area in Colombia declined from around 100 000 ha in the early 1990s to around 80 000 ha from 2000 onwards [26]. Coffee is planted at all altitudes, with the highest density of planting in Colombia in the ‘Coffee Cultural Landscape of Colombia’ World Heritage Site, also known as the ‘Coffee Triangle’ (figure 2*b*). The high-value *Coffea arabica* is grown primarily at altitudes from 1000 to 2000 m.a.s.l., while *C. robusta*, which has greater resistance to CLR, can be planted at lower altitudes [24] but is uncommon in Colombia. We defined a Region of Coffee in Colombia (RCC) as all pixels (averaged to the spatial resolution of JRA-55) with a harvested area greater than 1%, comprising around 267 000 km^2^.

### (b) Disease risk model

*Hemileia vastatrix* (Basidiomycota: Pucciniomycetes) is an obligate biotroph infecting *Coffea* spp., primarily attacking the leaves. CLR tends to reduce vigour and productivity of coffee plants, rather than kill them. Asexual disease progression occurs when the germinating uredospores form an appressorium (infection structure) and enter the host via the stomata [30]. Most stages of the CLR life cycle are strongly determined by weather [31]. Rain and wind assist spore dispersal, while germination is inhibited by solar radiation. As with many fungal foliar pathogens [7], germination of uredospores on the leaf surface and subsequent entry into via stomata requires the presence of liquid water and temperatures within a particular range [32–34]. Here, we focus on modelling the processes of spore germination and infection in relation to leaf wetness and temperature, in our estimation of disease risk.

Our model is motivated by a simple model of fungal foliar disease which assumes that infection by a germinated cohort of spores will take place if leaves have been wet for longer than a critical leaf wetness duration (*W*_crit_), with *W*_crit_ dependent upon both the pathogen-specific minimum wetness duration (*W*_min_) and the prevailing temperature via a temperature-dependent relative rate *r*(*θ*) determined by the cardinal temperatures—minimum (*θ*_min_), maximum (*θ*_max_) and optimum (*θ*_opt_)—of the pathogen [7]. The temperature response function requires estimates of the three cardinal temperatures [35]:
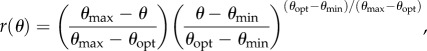
if *θ*_min_ ≤ *θ* ≤ *θ*_max_ and 0 otherwise, taking values in (0,1). The model is attractive because it requires relatively few biological parameters (cardinal temperatures and *W*_min_) and weather variables (leaf wetness and temperature).

The existing literature on temperature and water relations of CLR permit estimation of the required parameters for estimating disease risk [32–34]. While CLR germination and infection are strongly dependent upon the presence of liquid water (i.e. they will not occur under conditions of high humidity alone), one of the more recent studies suggests that the temperature response and infection processes of *H. vastatrix* are somewhat more complex than described in the aforementioned model. Specifically, the cardinal temperatures differ considerably between uredospore germination and appressorium formation [32], where appressorium formation is the prelude to infection through the stomata [30]. One interpretation of this ontogenetic change in temperature response is that *H. vastatrix* is adapted to maximize leaf wetness duration (LWD), germinating in the early evening and infecting overnight as temperatures cool, thereby avoiding dry spells during the day [32]. LWD and temperature measurements in coffee canopies in Costa Rica support this interpretation [36].

Another observation is that germination and appressorium formation (henceforth we term this latter stage ‘infection’ for convenience) are random processes, with probabilities varying through time [32], and therefore can be modelled using survival analysis [37]. The time *T* to transition (i.e. from a spore becoming wet to germination, or from germination to appressorium formation) is a random variable with probability density *f*(*t*) and cumulative distribution *F*(*t*) at time *t* after initiation. Our aim is to model *F*(*t*), which can be interpreted as the fraction of a population transitioned by *t*, for germination and infection by CLR (see the electronic supplementary material for details of the model and a worked example). *F*(*t*) is determined by the cumulative hazard function *H*(*t*), where the hazard function *h*(*t*) is the instantaneous risk of transformation given survival to *t*:
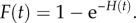


Conversely, the survival function *S*(*t*) = 1 − *F*(*t*). Survival processes are commonly modelled using the Weibull distribution which allows the hazard to vary over time:
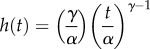

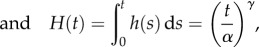
where *α* is the scale parameter, and *γ* the shape parameter (note, in some sources *γ* is the numerator of *t*). The processes of spore germination and infection are affected by temperature, hence we multiply *h*(*t*) and thus *H*(*t*) by *r*(*θ*), so that rates are greatest at the optimum temperature and decline to zero if outside the temperature range.

Jong *et al.* [32] provide mean values for the fraction of spores germinated, and fractions of germinated spores that have formed appressoria, at various time intervals, and at various temperatures (their fig. 1). We estimated parameters for the Weibull distributions and temperature response functions of germination and infection from the Jong *et al*. data by nonlinear least-squares optimization (see the electronic supplementary materials).

We assumed a constant rate of spore deposition and that ungerminated spores did not accumulate on leaves during dry periods, and so in each hour of a wet period a constant number of spores begin to germinate [7]. As the spore number is treated as unity, we omit this from the following description. If temperature were constant then calculation of *H*(*t*) would be straightforward, however, temperature varies arbitrarily over time and therefore *H*(*t*) must be calculated piecewise. Given an hourly time series, a wet period of length *W* will contain hourly intervals *i* = (1,…,*W*). For simplicity, let the relative rate *r_i_* during the *i*th interval be calculated from the mean of the temperatures at the beginning and end of the interval (*θ_i_*_−1_ + *θ_i_*)/2, such that
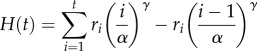
with parameters for germination or infection. A new cohort of spores begins to germinate at the beginning of each hour of each wet period, hence for the *j*th cohort
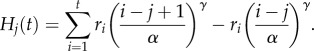
Thus, the *j*th cohort will begin to germinate at *t* = *j* − 1, and *H*(*t* < *j*) = 0. The total number of spores germinated during the *i*th hour is the sum across all cohorts
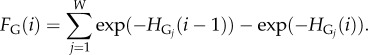
We use the same calculations, parametrized appropriately, for the number of germinated spores that infect, except that for the *k*th infecting cohort, the size of the starting population of germinated spores is *F*_G_(*k* − 1). This means that no infections occur in the first hour of a wet period. The final number of infecting spores is then the sum across all cohorts at the end of the wet period.

### (c) Climate data

The CLR model requires hourly estimates of temperature and leaf wetness. Air temperature is a commonly measured meteorological variable that varies relatively smoothly in time and space [38]. One potential issue in modelling disease risk is that pathogens are more likely to be controlled by the leaf temperature, rather than that of the surrounding air [39]. In contrast to temperature, leaf wetness is not usually measured by synoptic weather stations and shows complex spatio-temporal variability [38]. Leaves become wet via precipitation, fog or condensation (dewfall from the air or distillation from the soil), and dry out when relative humidity falls below saturation. Numerous methods exist for estimation of LWD, from detailed physical models of latent heat flux [40] to simpler statistical relationships with variables such as relative humidity [38,41,42].

The growing sophistication, spatio-temporal resolution and coverage of climate reanalyses potentially provide a new source of LWD estimates for plant pathology. Climate reanalyses combine multiple weather data sources with descriptions of the Earth system (e.g. soils and vegetation) and physical models of water and energy fluxes to provide regular, gridded estimates of the status of the oceans, atmosphere and land surface [22]. Here, we employ the Japanese 55-Year Reanalysis, JRA-55 [28], to drive our CLR risk model. We chose JRA-55 for several reasons: it has relatively high spatio-temporal resolution, it directly estimates variables of interest in plant pathogen risk modelling, and it is among the most sophisticated reanalyses currently available. JRA-55 provides global coverage of the period since 1958, with a temporal resolution of 3 h and spatial resolution of approximately 55 km. Data from ships, buoys, synoptic weather stations, radiosondes, balloons, aircraft and satellites are assimilated and processed using four-dimensional variational analysis (4D-Var). Outputs of relevance to pathogen risk modelling include canopy temperature and liquid water on the canopy surface. We obtained 3-hourly forecasts of canopy temperature and canopy liquid water for our ROI from 1990 to 2015 (http://rda.ucar.edu), and linearly interpolated these values to 1-hourly estimates. We assumed that any canopy moisture value above zero constituted a wet canopy, and ran our germination and infection models for the resulting wet periods in the R programming environment [43].

The model yielded relative numbers of infecting spores at the end of each wet period. For each pixel, we summed the relative infection numbers for each month from January 1990 to December 2015, and divided the sums by the number of days in the month to give a *mean daily infection risk* score per month. We investigated temporal variation in climate variables and disease risk by decomposing time series into seasonal, trend and residual components using the seasonal decomposition of time series by loess (STL) algorithm [27] implemented by function *stl* in R. We tested for significant temporal trends across our ROI using a generalized least-squares model, incorporating a spherical autocorrelation error model because of like spatial dependence among residuals, using the *gls* function in package *nlme* for R [44].

## 3. Results

**Figure 3.**
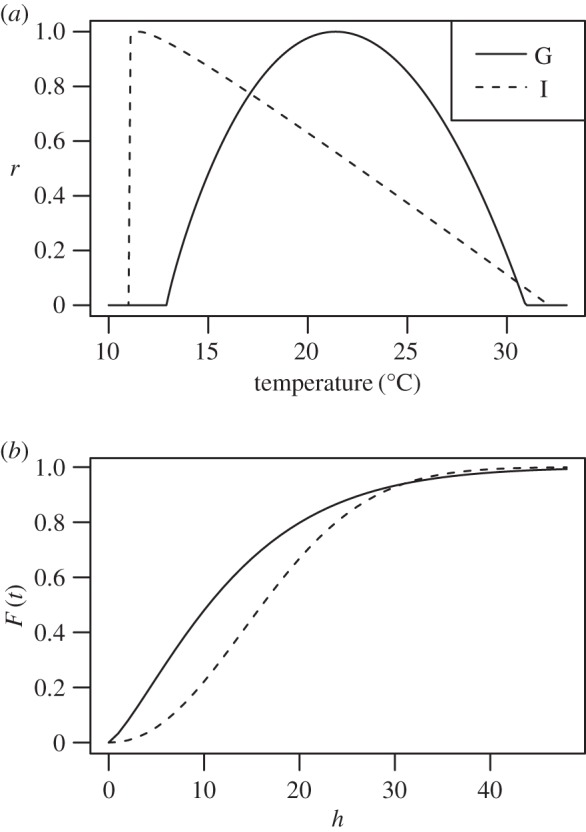
Model parameters. (*a*) Temperature response functions for germination (solid line) and appressorium formation (dashed line), from cardinal temperatures estimated from data in Jong *et al.* [32]. (*b*) Predicted germination (solid line) and infection (dashed line) trajectories over time at optimum temperature for the two processes.

From data in Jong *et al*. [32], we estimated that germination has *θ*_min_, *θ*_opt_ and *θ*_max_ as 12.9, 21.4 and 30.9°C, respectively, while appressorium formation was 11.6, 11.6 and 32.1°C. *θ*_min_ and *θ*_opt_ for appressorium formation were nearly identical, so we adjusted these slightly (11.0°C and 11.5°C) for the purposes of modelling. The relative rates of these processes therefore follow quite different responses to temperature (figure 3*a*). We estimated the parameters of the Weibull functions as *α* = 13.4 for germination and *α* = 19.1 for infection, and *γ* = 1.29 for germination and *γ* = 2.14 for infection (figure 3*b*).

**Figure 4.**
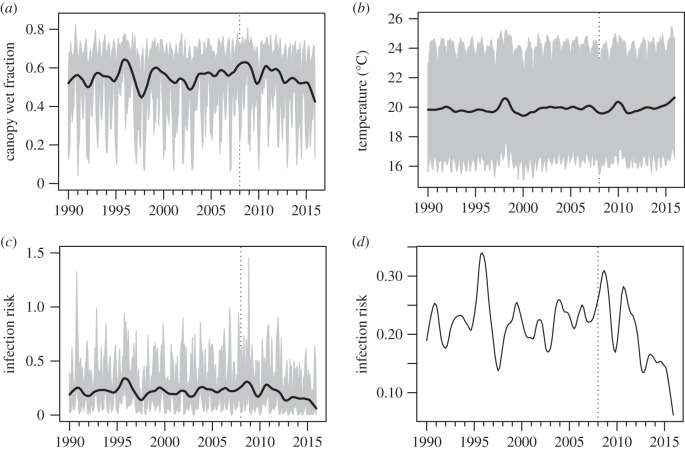
RCC climate and infection risk trends. (*a*) Fraction of the time canopy classified as wet (moisture above zero) in the RCC, 1990–2015. Grey shading indicates interquartile range of pixel values by month. Black line indicates trend component of median pixel values by month derived from seasonal-trend decomposition. (*b*) Canopy temperature during wet periods. (*c*) Mean daily infection risk. (*d*) Rescaled trend component of mean daily infection risk.

The maximum canopy water holding capacity in the JRA-55 model is approximately 0.5 mm, equivalent to 0.5 kg m^−2^. The temporal distribution of canopy water was strongly skewed, with values of zero occurring 54% of the time in the RCC. The median LWD (canopy water above zero) in the RCC was 17 h (interquartile range 11–17 h). The fraction of time the canopy was wet (i.e. including dry days) varied from around 0.2 to around 0.5 in the RCC, declining somewhat after 2012 (figure 4*a*). Canopy moisture was seasonal in the RCC, being lowest in January, rising to April, declining to July, then rising again to October. Canopy moisture followed a diurnal pattern of wetting in the early evening and drying in the morning (see the electronic supplementary material). The diurnal temperature cycle showed a minimum around 8.00 and rising to a maximum around 15.00, then declining more slowly over the afternoon and overnight. Mean daily canopy temperatures within the RCC showed no clear trend from 1990 to 2015, but increased during the El Niño–Southern Oscillation warming events of 1997–1998, 2009–2010 and 2015 (figure 4*b*).

**Figure 5.**
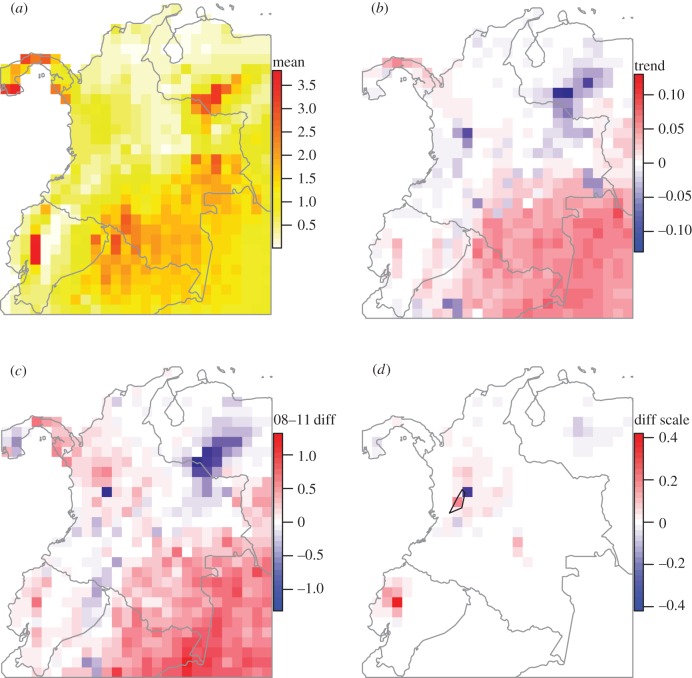
ROI infection risk. (*a*) Mean daily infection risk per pixel, 1990–2015. (*b*) Annual trend in mean daily infection risk 1990–2015. (*c*) Difference in mean daily infection risk between 2008 and 2011 and remaining years. (*d*) Difference in mean daily infection risk between 2008 and 2011 and remaining years, scaled by coffee area per pixel to visualize change in coffee-producing regions. The blue polygon encompasses the six UNESCO World Heritage Site areas within the Coffee Cultural Landscape of Colombia.

Mean daily infection risk per pixel from 1990 to 2015 was negatively correlated with coffee production across Colombia (Spearman rank correlation = −0.42), generally increasing towards the southeast, where little coffee is grown (figure 5*a*). The greatest mean risk was predicted in parts of Ecuador. Mean daily infection risk from 1990 to 2015 per pixel varied from zero to 3.82, with a median of 0.92, interquartile range 0.30–1.50. For reference, a value of one would occur if all spores in a cohort germinated, and subsequently infected a leaf. Mean daily infection risk per month was strongly correlated with mean canopy moisture per pixel (Spearman rank correlation = 0.87), and followed a similar seasonal cycle with a peak in October and minimum in January. The per pixel trend in infection risk from 1990 to 2015 over the ROI was small in comparison to the mean, with interquartile range 0.0021–0.034 yr^−1^, and increased towards the southwest where no coffee is produced (figure 5*b*). A similar pattern was seen in the difference between mean values from 2008 to 2011 and the remaining years, although northeastern Colombia and Ecuador also had somewhat elevated predicted risk (figure 5*c*).

Scaling the 2008–2011 difference by the relative amount of coffee grown per pixel suggested elevated infection risk during this period in the RCC and Ecuador (figure 5*d*). In the RCC, mean daily infection risk was somewhat elevated in 2008–2011, declining again after 2011, but was also high in 1996 (figure 4*c*,*d*). Taking spatial autocorrelation among pixels into account, no statistically significant linear trend in mean daily infection risk was detected from 1990 to 2015 in the RCC (generalized least-squares model, mean −0.00074 ± 0.0025, d.f. = 68, *t* = −0.29, *p* = 0.76). However, a small but significant elevation in mean daily infection risk between 2008 and 2011 compared with other years was detected (generalized least-squares model, mean difference in 2008–2011 = 0.055 ± 0.024, d.f. = 68, *t* = 2.25, *p* = 0.0274).

## 4. Discussion

Fungal pathogen life cycles are strongly determined by weather, particularly temperature and water availability. Obtaining estimates of water availability is problematic and remains an issue for disease prediction [38]. We have proposed that the increasing observational data assimilation, modelling sophistication, spatio-temporal resolution and coverage of climate reanalyses offer a powerful, but underused, tool to assist in modelling historical and current fungal plant pathogen risk. We illustrated this approach by testing the hypotheses that (i) the weather was responsible for a recent outbreak of CLR in Colombia and (ii) that climate change increased the probability of weather conditions favourable to CLR, by using the state-of-the-art JRA-55 reanalysis to drive a model of spore germination and infection parametrized using experimental data. While CLR infection risk was elevated in 2008–2011 in coffee-growing regions of Colombia, we found no compelling evidence for a large increase in predicted infection risk over the period in which the CLR outbreak is reported to have been most severe, and no long-term trend in risk from 1990 to 2015. Therefore, we conclude that while weather conditions in 2008–2011 may have slightly increased the predicted risk of CLR infection, long-term climate change is unlikely to have increased disease risk. We found a decline in mean daily LWD from around 2012–2015 and a resulting decline in daily CLR risk, suggesting that weather conditions have become less favourable for CLR in recent years. It is possible that this drying helped to bring the epidemic to a close.

Early indicators suggest that new aggressive variants of *H. vastatrix* did not emerge to cause the recent CLR epidemic [45]. This, however, merits greater investigation with comparative genomic analysis gathered across the region. Another analysis of the outbreak concluded that changes in coffee management exacerbated the impact of CLR on production [23]. In particular, fertilizer use declined due to dramatic price rises during the 2008 global financial crisis, leading to decreased vigour of coffee plants. The same study suggested that increased annual rainfall, reduced sunshine and decreased diurnal temperature range favoured infection and reduced the latent period of infection. While precipitation can indicate when plant canopies will be wetted, LWD is dependent not only on input by precipitation, but also by condensation and the rate of evaporation. Hence, models of energy and moisture fluxes between the soil, the plant canopy and the atmosphere are required to fully describe the dynamics of LWD [40]. Climate reanalyses can provide plant pathologists with a convenient estimate of LWD without the need for data assimilation from multiple sources, and complex atmospheric physics models. However, hydrology is among the most difficult components of the climate system to model, and caution must be applied when using hydrological outputs of reanalyses [28]. Thus, relative differences in canopy moisture among pixels and over time are likely to be more informative than the absolute values. Reanalyses provide more biologically relevant temperature measures, such as canopy temperature, for modelling [39]. This is important because leaf temperature can differ significantly from air temperature, particularly under water stress conditions [46]. Although the air-canopy temperature difference will be minimal at night when canopies are wet and the infection process takes place, further research is required into the effect of choosing different temperature estimates on disease risk estimates.

The approach we employed has been termed ‘forward’ modelling in the species distribution modelling literature, whereby process-based models of a species' life history are developed independently of any calibration against observational data of occurrence or abundance [47]. This contrasts with correlative models which statistically relate environmental variables to species abundance, and in which there is an implicit assumption that biological processes are responding to the environment. The parameters of process-based models may be tuned using observational data, but in purely forward models like ours, parameters are derived independently of observations, i.e. from controlled experiments. Forward process-based models avoid the problem of equifinality, whereby different parametrizations yield similar predictions. Forward models can be used to test hypotheses concerning responses of species to environmental change because they are less likely to produce correct results for the wrong reasons, in contrast to correlative models parametrized from observational data [47]. An additional reason for parametrizing models from experimental data, rather than observed distributions, is that observed distributions do not reflect the fundamental climatic niche of a species but rather a subset constrained by biotic factors and migration [48,49]. In our model, the cardinal temperatures are abstractions of an axis of the climatic niche. Process-based models should therefore be parametrized from experimental data or from biophysical first principles if the fundamental niche is to be estimated [50].

A weakness of our approach is that we modelled only two of the various life cycle components affected by weather. We omitted the processes of spore production, and dissemination by rain, wind and non-climatic factors such as disturbance of the canopy during management and harvesting activities [31]. Thus, our conclusions are limited to considerations of the probabilities of leaf infection. Wind speed and direction could be obtained from climate reanalyses and used to model long-range dispersal, if the shape of the dispersal kernel were known [51]. However, there are currently insufficient published empirical data to enable reliable parametrization of these processes. Short-range dispersal by rain splash or management occurs at spatial scales far below the resolution of current reanalyses.

Spore germination and appressorium formation have different temperature response functions [32]. Several fungi have different temperature optima for germination and growth [52], but this is not considered in the simple model [7] upon which we based our analysis. Parameters for these response functions were estimated from a single study [32], which to our knowledge has not been replicated. This lack of replication may be because *H. vastatrix* is an obligate biotroph, and therefore it cannot be cultured *in vitro*, making experimental manipulation more difficult. Further investigation of temperature responses, including acclimation and adaptation potential, is warranted to improve the parametrization of CLR risk models.

We modelled infection risk in the absence of consideration of the host response to varying weather conditions, and the role of the host in disease epidemiology, e.g. as a source of inoculum. Coffee yields in Colombia have been highly erratic over time (figure 1), a consequence of varying weather, disease pressure, management and socio-economic factors. While the decline in yield beginning in 2008 is clear, large yield fluctuations occurred in the 1990s, and attempts have been made to explain variability *post hoc* with reference to various events and trends [23]. In order to properly understand the likely impact of climate change on production, and partition out the effects of weather, disease and other factors on coffee yield, a weather-driven coffee yield model is required. Simultaneous modelling of crop yield and disease risk has been undertaken, for example, for potato late blight, and used to project future disease risks [17]. Unfortunately, coffee yield models appear to be under-developed in the literature, for example, the UN FAO AQUACROP model has not been parametrized for coffee [53].

For CLR and many other fungal plant diseases, temperature and leaf wetness are the most important determinants of infection risk [7]. The JRA-55 reanalysis directly estimates relevant variables—canopy temperature and surface water—at a temporal resolution sufficient to enable hourly time series to be used in disease models. Other global reanalyses, such as MERRA and ERA-Interim, do not directly estimate these variables. While JRA-55 is among the most sophisticated and data-rich sources currently available, there remain areas for improvement from a disease modelling perspective. In common with many reanalyses, precipitation observations are not incorporated to drive the analysis, but rather precipitation is predicted from atmospheric temperature and humidity [28,54]. Another potential climate data source, the North American Regional Reanalysis, does assimilate precipitation observations, and it has a number of other attractive features: temporal resolution is 3 h, spatial resolution is slightly finer (approx. 32 km) than JRA-55, and canopy surface water is estimated [55,56]. However, upon investigation we found that observational data sources affecting the hydrological cycle changed in 2003, severely biasing results in our ROI. The spatial resolution of JRA-55 is finer than previous reanalyses, but remains coarse relative to the biological systems we wish to model. Topography, microclimate and land use can vary greatly within a 55 km grid cell. However, the increasing availability of high-resolution satellite data and growing computing power promise to continually improve the data available for modelling plant disease. In addition, the relationship between mean canopy moisture across a grid cell and the spatial distribution of surface moisture within a canopy, and its impact on disease development, is unknown.

Climate reanalysis is the task of the climate research community, but responsibility for recording disease outbreaks, which are the observational data against which models can be tested, lies with the producers, researchers, agribusinesses and plant protection agencies that monitor agricultural systems. It is evident that despite the threat posed by pathogens, serious data gaps remain of where and when outbreaks occur, particularly in the developing world [21]. Platforms such as the IPM PIPE disease monitoring network in the USA, the AHDB monitoring service in the UK, and the observations of CLR provided by the National Coffee Association in Guatemala [23], serve as examples of the kinds of data required for model validation. Often, short-term, small-scale observations used to validate models published in the scientific literature are not deposited in open-access databases to enable re-use by other researchers. We hope that new initiatives such as Global Open Data for Agriculture and Nutrition will begin to address this issue, bringing plant pathology data into step with the open-access philosophy seen in climate research.

## Supplementary Material

Supplementary Material
