# Supplementary material for: Modelling coffee leaf rust risk in Colombia with climate reanalysis data
Source: Philos Trans R Soc Lond B Biol Sci. 2016 Dec 5;371(1709):20150458. doi: 10.1098/rstb.2015.0458 (PMC5095537; doi:10.1098/rstb.2015.0458)
Supplement: Supplementary Material [file rstb20150458supp1.pdf]

Supplementary Material

Modelling coffee leaf rust risk in Colombia with climate reanalysis data

Daniel P. Bebber, Angela Delgado Castillo & Sarah J. Gurr

September 2016

Model

Infection of plant leaves by fungal pathogens generally occurs in two stages. First, spores on the leaf surface germinate to produce a germ tube, and then these germ tubes infect the leaf by penetrating the leaf surface. Spore germination and infection can be modelled as survival processes, with transitions from spore to germinated spore, and germinated spore to infection event. In *survival analysis*, the time T to transition is a random variable in $(0, \infty)$ with probability density $f(t)$ and cumulative distribution $F(t)$ at time t after initiation. Our aim is to model $F(t)$ for germination and infection by coffee leaf rust. The probability of surviving in the untransformed state until t is known as the *survival function*:

$$S(t) = 1 - F(t) = Pr(T > t) \tag{1}$$

The instantaneous risk of changing state at time t, given survival until t, is known as the *hazard function*:

$$h(t) = \lim_{\Delta t \to 0} Pr(t \leq T < t + \Delta t | T \geq t)/\Delta t \tag{2}$$

The cumulative hazard $H(t) = \int_0^t h(s)ds$ is related to survival by $S(t) = e^{-H(t)}$. We can therefore calculate the expected fraction of potential transitions that have occurred by t from the area under the curve $h(t)$:

$$F(t) = 1 - S(t) = 1 - e^{-H(t)} \tag{3}$$

The Weibull distribution is commonly used to model systems in which $h(t)$ varies with t (compared with constant $h(t)$ in the exponential distribution). The Weibull distribution is defined by two parameters, the scale α and the shape γ:

$$h(t) = \left(\frac{\gamma}{\alpha}\right)\left(\frac{t}{\alpha}\right)^{\gamma-1} \tag{4}$$

$$H(t) = \left(\frac{t}{\alpha}\right)^{\gamma} \tag{5}$$

where α and γ may differ for germination and infection.

In many foliar fungal pathogens, both germination and infection require liquid water on the leaf surface. Let W by the leaf wetness duration. Germination and infection begin when the leaf becomes wet (at $t = 0$) and cease when the leaf dries again (at $t = W$). In the following notation, subscript G refers to germination, and I to infection. The total time from the leaf becoming wet to infection $T_{\text{tot}} = T_G + T_I$. Because drying of the leaf surface will kill germinated spores, the leaf wetness duration $W \geq T_{\text{tot}}$ for successful infection. The rate of fungal spore germination and infection varies not only with t, but also with temperature θ. We modelled the response using a beta function growth model (Yan & Hunt 1999). At the optimum temperature of the process θ_{opt} the relative rate $r(\theta) = 1$, and $r(\theta)$ declines to zero as θ increases towards the maximum temperature θ_{max} or decreases towards the minimum temperature θ_{min} for the process:

$$r(\theta) = \left(\frac{\theta_{\text{max}} - \theta}{\theta_{\text{max}} - \theta_{\text{opt}}}\right) \left(\frac{\theta - \theta_{\text{min}}}{\theta_{\text{opt}} - \theta_{\text{min}}}\right)^{\frac{\theta_{\text{opt}} - \theta_{\text{min}}}{\theta_{\text{max}} - \theta_{\text{opt}}}} \tag{6}$$

when $\theta_{\text{min}} < \theta < \theta_{\text{max}}$ and $r(t) = 0$ otherwise. We scale the hazard function by r:

$$h(\theta, t) = r(\theta) \left(\frac{\gamma}{\alpha}\right) \left(\frac{t}{\alpha}\right)^{\gamma - 1} \tag{7}$$

Therefore if θ and thus r were constant:

$$H(\theta, t) = r(\theta) \left(\frac{t}{\alpha}\right)^{\gamma} \tag{8}$$

However, θ varies arbitrarily with t, precluding a simple expression for $\theta(t)$ and thus for $\int r(t)h(t)dt$. $H(t)$ must therefore be calculated piecewise in t, i.e. by summing $H(t)$ over time intervals during which r can be expressed as a function of t. Climate variables in the JRA-55 dataset given at 3-hourly intervals were linearly interpolated to hourly estimates, therefore we integrated piecewise over these hourly intervals. Let i be an integer $(1, ..., W)$ denoting time in hours since the start of a wet period, at which points we want to evaluate the number of spores that have germinated and infected. First we modelled a linear change in θ over time within each hourly interval:

$$\theta(t) = \theta_{i-1} + \frac{\theta_i - \theta_{i-1}}{t_i - t_{i-1}}(t - t_{i-1}) \tag{9}$$

where $i - 1 < t \leq i$. Let $c_1 = (\theta_i - \theta_{i-1})/(t_i - t_{i-1})$ and $c_2 = \theta_{i-1} - c_1 t_{i-1}$ giving

$$\theta(t) = c_2 + c_1 t \tag{10}$$

and so

$$r(t) = \left(\frac{\theta_{\max} - c_2 - c_1 t}{\theta_{\max} - \theta_{\text{opt}}}\right)\left(\frac{c_2 + c_1 t - \theta_{\min}}{\theta_{\text{opt}} - \theta_{\min}}\right)^{\frac{\theta_{\text{opt}} - \theta_{\min}}{\theta_{\max} - \theta_{\text{opt}}}} \tag{11}$$

Let $c_3 = \theta_{\max} - c_2$, $c_4 = c_2 - \theta_{\min}$, $c_5 = \theta_{\max} - \theta_{\text{opt}}$, $c_6 = \theta_{\text{opt}} - \theta_{\min}$ and $c_7 = c_6/c_5$ giving

$$r(t) = \left(\frac{c_3 - c_1 t}{c_5}\right)\left(\frac{c_4 + c_1 t}{c_6}\right)^{c_7} \tag{12}$$

We attempted integration by parts on $\int r(t)h(t)dt$, but this did not yield an analytical solution. Therefore, we simplified the model by assuming constant temperature over the interval $(i-1, i]$ as $\theta_{i-1,i} = (\theta_{i-1} + \theta_i)/2$, making r_i constant in each hourly interval. Assume cohorts of spores, each of equal size N, begin to germinate at the start of each wet hour. We let $N = 1$ and hence can omit N from the description. Let j be an integer vector $(1, ..., W)$ denoting each cohort, with the initiation of each cohort at $t = j - 1$. The cumulative hazard function for germination of the jth cohort is the sum of the cumulative hazards across all hourly intervals:

$$H_{G,j}(t) = \sum_{i=1}^{t} r_{Gi}.H_G(i - j + 1) - r_{Gi}.H_G(i - j) \tag{13}$$

for $j \geq i$ and $H_{G,j}(i) = 0$ otherwise. The total number of spores germinated in the hourly interval $t = (i - 1, i)$ is the sum of the fraction germinated in the interval across all cohorts:

$$F_G(i) = \sum_{j=1}^{W} 1 - \exp(-H_{G,j}(i)) - (1 - \exp(-H_{G,j}(i-1))) \tag{14}$$

$$= \sum_{j=1}^{W} \exp(-H_{G,j}(i-1)) - \exp(-H_{G,j}(i)) \tag{15}$$

We modelled the process of infection similarly. Let k be an integer $(1, ..., W)$ denoting each infecting cohort, with initiation of each cohort at $t = k - 1$. The initial size of the kth cohort is the total number of spores germinated during the hourly interval $t = (k - 1, k - 1)$, i.e. $F_G(k - 1)$. This means that no infections occur during the first hour, because $F_G(0) = 0$, an unavoidable result of the need to calculate F_G and F_I piecewise. The number of infections of the kth cohort is:

$$F_{I,k}(t) = F_G(k - 1)(1 - \exp(-H_{I,k}(t))) \tag{16}$$

The total number of infections at the end of the wet period is:

$$F_I(W) = \sum_{k=1}^{W} F_{I,k}(t) \tag{17}$$

Numerical example

Data

We used the R programming environment to model the germination and infection processes. We illustrate our analysis below with an example of one wet period in a coffee-growing area of Colombia. The selected pixel is centered on 74.812°E 4.212°N, extending from 75.093 to 74.531°E and 3.931 to 4.493°N. Mean altitude is 451m, and coffee area 3.9%. JRA-55 data are given at 3-hourly time steps in UTC time, hence we convert to local time (-5 hours) for plotting.

```
load("JRA55 eg.Rdata") #Load the data
#convert hour to POSIXct, -5hr for Colombia
dat$date <- as.POSIXct(dat$hour*3600, origin = "1800-01-01 00:00:00",
                       tz = "America/Bogota")
head(dat, 5) #Inspect variables
```

```
##         lon   lat  id    hour       cm    ct                date
## 390  -74.81 4.212 210 1577829 0.003427 22.94 1979-12-31 16:00:00
## 1227 -74.81 4.212 210 1577832 0.000000 21.60 1979-12-31 19:00:00
## 2064 -74.81 4.212 210 1577835 0.000000 19.22 1979-12-31 22:00:00
## 2901 -74.81 4.212 210 1577838 0.000000 17.85 1980-01-01 01:00:00
## 3738 -74.81 4.212 210 1577841 0.000000 18.33 1980-01-01 04:00:00
```

The first column is the index in the full dataset, `hour` is the hour since midnight on 1st January 1800, `cm` is canopy moisture in kg m^{-2}, `ct` is canopy temperature in °C converted from K in the JRA55 dataset, and date is `hour` converted to POSIXct format using function `strftime()`.
We selected the first week of January 2008 for analysis.

```
jan <- subset(dat, date >= "2008-01-01 00:00:00" & date < "2008-01-08 00:00:00")
# Plot canopy moisture and temperature
require(ggplot2, quietly = TRUE)
theme_set(theme_bw(base_size = 10))
ggplot(data = jan, aes(x = date, y = cm)) + geom_line() + geom_point() + xlab(NULL)
```

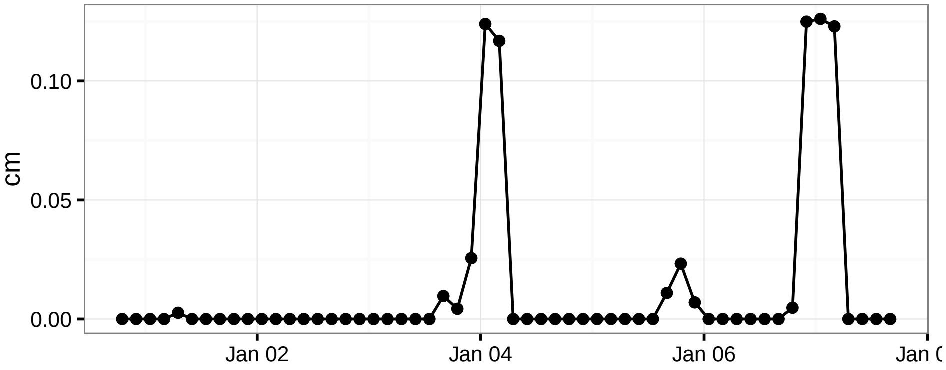

```r
ggplot(data = jan, aes(x = date, y = ct)) + geom_line() + geom_point() + xlab(NULL)
```

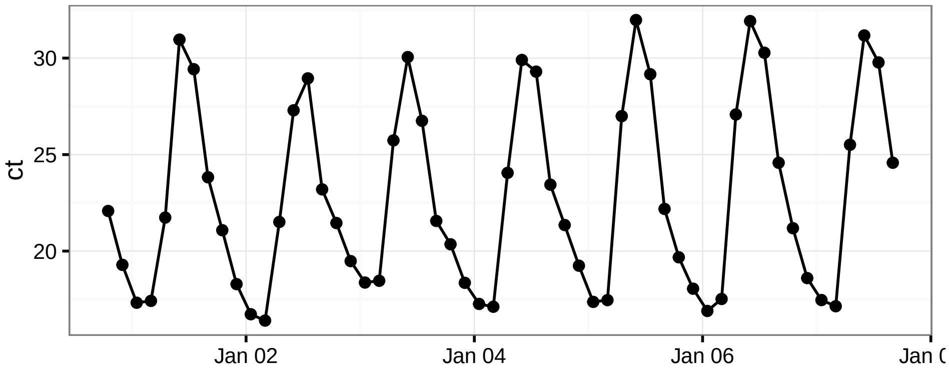

The question of what constitutes a wet canopy is illustrated in the data. There are transient wet periods on 1st and 6th January, with longer and wetter periods on the 4th and 7th. Wet periods coincide with cooling overnight canopy temperatures. We selected the first of the longer wet periods for analysis. The canopy begins to become moist after 18:00 on 3rd Jan, but the value is very low and the canopy dries slightly before wetness increases again at midnight. The maximum wetness is around 0.12 kg m^{-2}, considerably below the maximum of around 0.5 kg m^{-2} in the full dataset. We interpolated `cm` and `ct` linearly using `interpTs()` in package **wq**, to give hourly estimates of wetness and temperature:

```r
require(wq, quietly = TRUE)
hr0 <- seq(min(jan$hour), max(jan$hour))  #vector of hours
cm0 <- rep(NA, length(hr0))  #vector for hourly moisture
ct0 <- rep(NA, length(hr0))  #vector for hourly temperature
cm0[hr0 %in% jan$hour] <- jan$cm  #assign known moisture values
ct0[hr0 %in% jan$hour] <- jan$ct  #assign known temperature values
cm0 <- interpTs(cm0, type = "linear")  #interpolate moisture
ct0 <- interpTs(ct0, type = "linear")  #interpolate temperature
date0 <- as.POSIXct(hr0 * 3600, origin = "1800-01-01 00:00:00")
jan0 <- data.frame(date = date0, hr = hr0, cm = cm0, ct = ct0)  #store in data.frame
rm(hr0, cm0, ct0, date0)  #clean up workspace
# Inspect some hourly values...
```

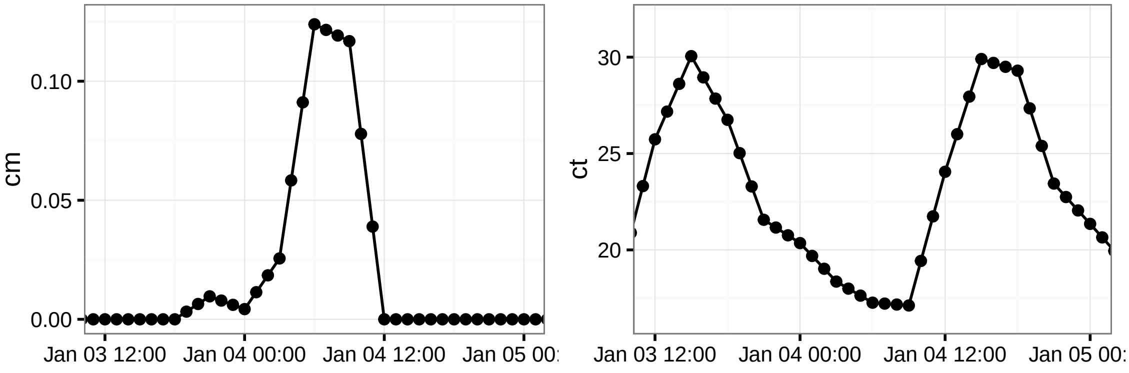

We took any moisture above zero to indicate a wet canopy, and identified wet periods using `rle2()`

in package **accelerometry**. `rle2` is faster and more versatile than `rle()` in base R, giving the start and end indices, and lengths, of runs of identical values.

```
require(accelerometry, quietly = TRUE)
periods <- rle2(as.numeric(jan0$cm > 0), indices = TRUE)
periods  #output is a matrix, not data.frame!
```

```
##       values starts stops lengths
##  [1,]      0      1    10      10
##  [2,]      1     11    15       5
##  [3,]      0     16    67      52
##  [4,]      1     68    84      17
##  [5,]      0     85   115      31
##  [6,]      1    116   126      11
##  [7,]      0    127   142      16
##  [8,]      1    143   156      14
##  [9,]      0    157   166      10
```

```
wet <- periods[periods[, 1] == 1, ]  #select only the wet periods
wet0 <- jan0[wet[2, 2]:wet[2, 3], ]  #select the first wet period
wet0
```

```
##                   date      hr       cm    ct
## 68 2008-01-03 19:00:00 1823347 0.003219 25.02
## 69 2008-01-03 20:00:00 1823348 0.006437 23.29
## 70 2008-01-03 21:00:00 1823349 0.009656 21.56
## 71 2008-01-03 22:00:00 1823350 0.007868 21.16
## 72 2008-01-03 23:00:00 1823351 0.006080 20.76
## 73 2008-01-04 00:00:00 1823352 0.004292 20.35
## 74 2008-01-04 01:00:00 1823353 0.011384 19.69
## 75 2008-01-04 02:00:00 1823354 0.018477 19.02
## 76 2008-01-04 03:00:00 1823355 0.025570 18.36
## 77 2008-01-04 04:00:00 1823356 0.058353 17.99
## 78 2008-01-04 05:00:00 1823357 0.091136 17.63
## 79 2008-01-04 06:00:00 1823358 0.123918 17.26
## 80 2008-01-04 07:00:00 1823359 0.121554 17.21
## 81 2008-01-04 08:00:00 1823360 0.119189 17.16
## 82 2008-01-04 09:00:00 1823361 0.116825 17.12
## 83 2008-01-04 10:00:00 1823362 0.077883 19.43
## 84 2008-01-04 11:00:00 1823363 0.038942 21.74
```

We now have the data required for modelling the germination and infection processes over time.

Estimating model parameters

We used the germination and appressorium formation data in Jong et al. (1987) to estimate parameters for the temperature response function r, and Weibull parameters α and γ of the models

for F_G and F_I. The data were manually abstracted from Fig. 1 in Jong et al. (1987) using pixel coordinates in a digital image of the figure. G and I are the measured fractions of germinated spores, and germinated spores that have formed appressoria (infection structures), respectively.

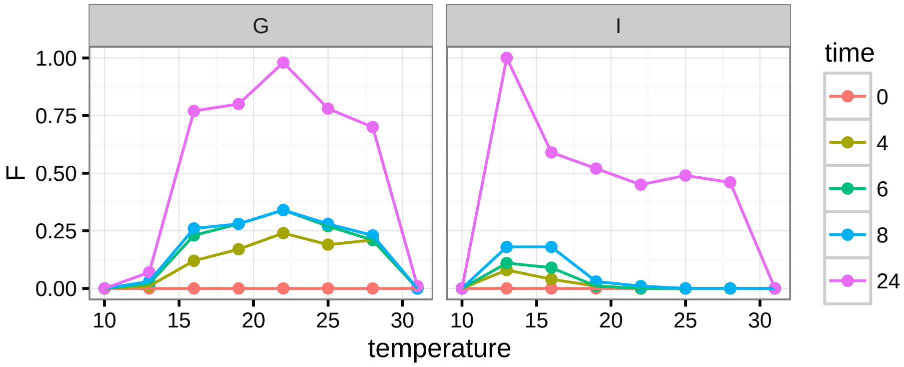

We optimized $\theta_{\min}$, θ_{opt}, $\theta_{\max}$, α and γ by minimizing $\sum(F(\theta,t) - y(\theta,t))^2$ where y is the data, for germination and infection. The optimization algorithm requires initial parameter values, which we estimated by eye from the data and some preliminary fits.

```r
# Function for r(theta)
rtheta <- function(theta, tmin, topt, tmax) {
    r <- ((tmax - theta)/(tmax - topt)) * ((theta - tmin)/(topt - tmin))^((topt -
        tmin)/(tmax - topt))
    r[theta <= tmin | theta >= tmax] <- 0  #set values outside temperature range to zero
    r
}
# Function for F(t) where is par = c(tmin, topt, tmax, alpha, gamma)
Ft <- function(time, theta, par) {
    r <- rtheta(theta, par[1], par[2], par[3])
    1 - exp(-r * (time/par[4])^par[5])
}
# Function to calculate sums of squares
F.opt <- function(par, theta, time, y) {
    sum((Ft(time, theta, par) - y)^2)
}
# Initial parameter estimates
parG <- c(12, 22, 31, 10, 2)
parI <- c(10, 13, 31, 15, 2)
# Optimization using the Nelder-Mead algorithm
optG <- optim(parG, F.opt, time = jong$time, theta = jong$temp, y = jong$G)$par
optI <- optim(parI, F.opt, time = jong$time, theta = jong$temp, y = jong$I)$par
names(optG) <- names(optI) <- c("tmin", "topt", "tmax", "alpha", "gamma")
optG
```

```
##   tmin   topt   tmax  alpha  gamma
## 12.909 21.410 30.943 13.363  1.287
```

```
optI
```

```
##   tmin   topt   tmax  alpha  gamma
## 11.570 11.570 32.109 19.121  2.141
```

```
optI[1:2] <- c(11, 11.5)  #adjust tmin and topt for infection
# plot data and model fits...
```

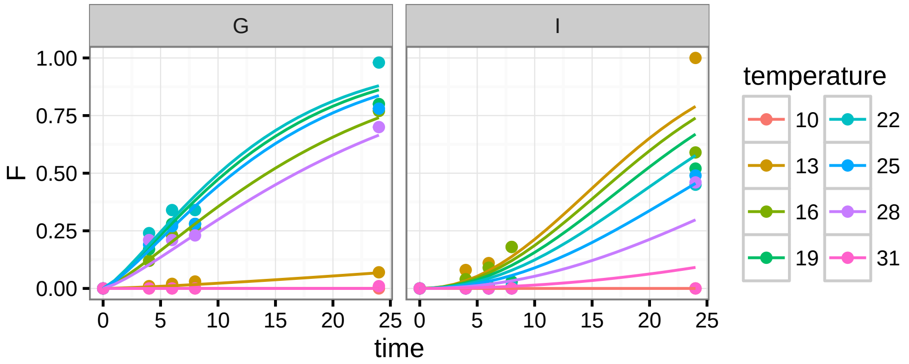

R^2 for the fitted germination model was 0.979, and for infection 0.911. The plots show the poor distribution of sampling over time in Jong et al., with no measurements between 8 and 24 hours. However, the fits are a reasonable approximation of the measurements and so we can apply our models to the example wet period. The only adjustment we made to the optimized parameters was to θ_{min} for infection, which was very close to θ_{opt}. The data and models suggest that both germination and infection will be relatively low over the example 7 h wet period.

Despite the large difference in θ_{opt} for germination and infection, r_G and r_I are strongly correlated over time, greatest overnight (when wet periods occur) and lowest during the late afternoon when temperatures reach 30°C.

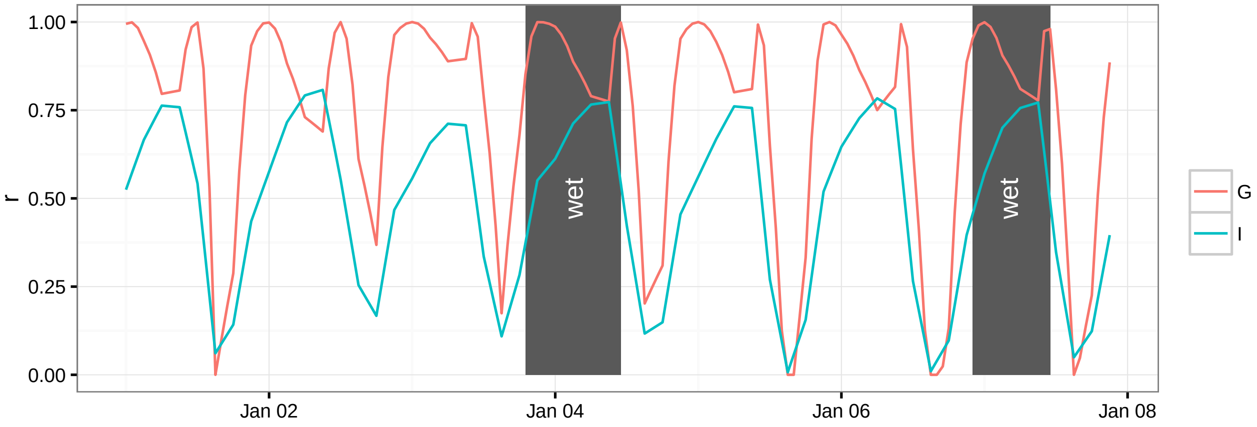

Modelling germination and infection

We estimated F_G and F_I piecewise by hour, modelling the temperature during each hour as the mean of the temperatures at the beginning and end of each hourly interval, so that r_G and r_I vary stepwise with t:

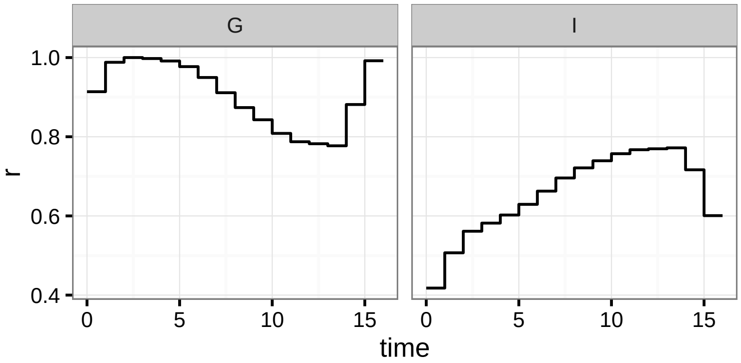

The hazard therefore also varies discontinuously with t. We calculated the cumulative hazard $H(t)$ through the wet period for each hourly cohort. The pieces making up $H(t)$ are equal to the areas of the coloured bars:

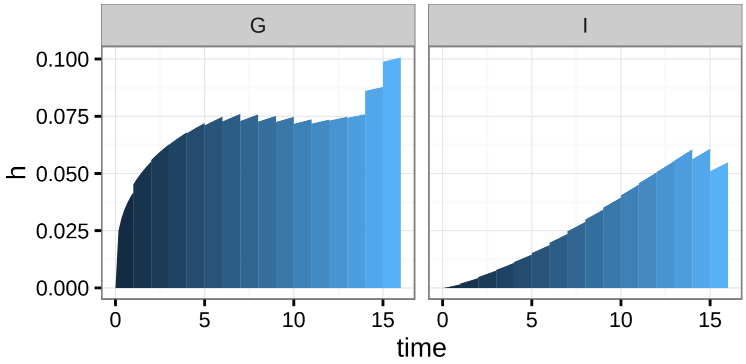

Loops are slower than matrix operations in R so we constructed $i \times j$ matrices to calculate $H(t)$ for each hourly interval and each cohort. In the following we show only a subset of the values for illustration:

```
H <- function(ct, par) {
    # takes canopy temperature as input
    w <- length(ct) - 1  #length of wet period
    ti <- matrix(ct[1:w] + diff(ct)/2, nc = w, nr = w, byrow = F)  #mean temperatures
    ri <- rtheta(ti, par[1], par[2], par[3])  #relative rates
    im <- matrix(1:w, nc = w, nr = w) - rep((1:w) - 1, each = w)  #end time
    im0 <- im - 1  #start time
    Hm <- ri * ((im/par[4])^par[5] - (im0/par[4])^par[5])
    Hm[is.na(Hm)] <- 0  #replace NA with zeros when j < i
    dimnames(Hm) <- list(i = 1:w, j = 1:w)
    Hm  #returns matrix giving H for each cohort and interval
}
# H for germination
HG <- H(wet0$ct, optG)
HG[1:4, 1:4]  #showing only first four rows and columns
```

```
##    j
## i         1       2       3       4
##   1 0.03247 0.00000 0.00000 0.00000
```

```
##   2 0.05058 0.03510 0.00000 0.00000
##   3 0.05943 0.05119 0.03553 0.00000
##   4 0.06535 0.05929 0.05107 0.03544
```

```
# Cumulative hazard across intervals
HGc <- apply(HG, 2, cumsum)
HGc[1:4, 1:4]
```

```
##    j
## i         1       2       3       4
##   1 0.03247 0.00000 0.00000 0.00000
##   2 0.08304 0.03510 0.00000 0.00000
##   3 0.14247 0.08629 0.03553 0.00000
##   4 0.20782 0.14558 0.08659 0.03544
```

```
# F(t) for each cohort
FG <- 1 - exp(-HGc)
FG[1:4, 1:4]
```

```
##    j
## i         1       2       3       4
##   1 0.03194 0.00000 0.00000 0.00000
##   2 0.07969 0.03449 0.00000 0.00000
##   3 0.13279 0.08267 0.03490 0.00000
##   4 0.18765 0.13548 0.08295 0.03482
```

```
# Hourly fractional germination per cohort
FGc <- apply(rbind(0, FG), 2, diff)
FGc[1:4, 1:4]
```

```
##         1       2       3       4
## 1 0.03194 0.00000 0.00000 0.00000
## 2 0.04774 0.03449 0.00000 0.00000
## 3 0.05310 0.04818 0.03490 0.00000
## 4 0.05486 0.05281 0.04805 0.03482
```

```
# Total hourly germination
FGi <- rowSums(FGc)
FGi[1:4]
```

```
##       1       2       3       4
## 0.03194 0.08224 0.13618 0.19054
```

The calculations above give us the relative total number of germinated spores at each hour after the beginning of the wet period. We have assumed that the number of spores N that are available to germinate at the beginning of each hour is constant, and so N drops out of the calculations. We use similar calculations for the infection process.

```r
# H for infection
HI <- H(wet0$ct, optI)
HI[1:4, 1:4]
```

```
##    j
## i           1         2        3        4
##   1 0.0007546 0.0000000 0.000000 0.000000
##   2 0.0031215 0.0009156 0.000000 0.000000
##   3 0.0061790 0.0034571 0.001014 0.000000
##   4 0.0093959 0.0064046 0.003583 0.001051
```

```r
# Cumulative hazard across intervals
HIc <- apply(HI, 2, cumsum)
HIc[1:4, 1:4]
```

```
##    j
## i           1         2        3        4
##   1 0.0007546 0.0000000 0.000000 0.000000
##   2 0.0038762 0.0009156 0.000000 0.000000
##   3 0.0100551 0.0043726 0.001014 0.000000
##   4 0.0194510 0.0107773 0.004597 0.001051
```

```r
# F(t) for each cohort
FI <- 1 - exp(-HIc)
FI[1:4, 1:4]
```

```
##    j
## i           1         2        3       4
##   1 0.0007543 0.0000000 0.000000 0.00000
##   2 0.0038687 0.0009151 0.000000 0.00000
##   3 0.0100047 0.0043631 0.001013 0.00000
##   4 0.0192631 0.0107194 0.004587 0.00105
```

```r
# Size of cohorts of germinated spores, FG(t=0) = 0
FG0 <- matrix(c(0, FGi)[1:length(FGi)], nr = 16, nc = 16, byrow = T)
FG0[1:4, 1:4]
```

```
##      [,1]    [,2]    [,3]   [,4]
## [1,]    0 0.03194 0.08224 0.1362
## [2,]    0 0.03194 0.08224 0.1362
## [3,]    0 0.03194 0.08224 0.1362
## [4,]    0 0.03194 0.08224 0.1362
```

```r
# Multiply by available number of germinated spores
FI_final <- FG0 * FI
FI_final[1:4, 1:4]
```

```
##    j
## i   1         2         3         4
##   1 0 0.000e+00 0.000e+00 0.0000000
##   2 0 2.923e-05 0.000e+00 0.0000000
##   3 0 1.394e-04 8.334e-05 0.0000000
##   4 0 3.424e-04 3.772e-04 0.0001431
```

```r
# FI at end of wet period
sum(FI_final[16, ])
```

```
## [1] 0.3914
```

```r
# Plot FI(t)...
```

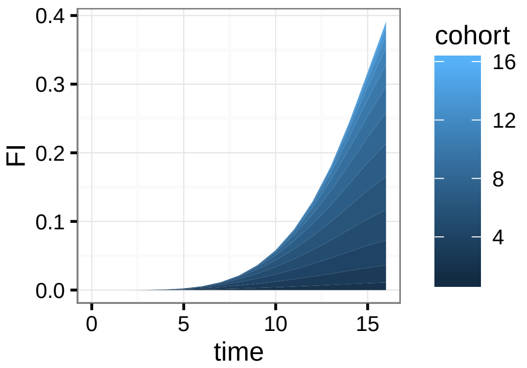

The model suggests that over the 16 h wet period we selected, the total infection would amount to around 39% of N, our notional hourly spore cohort size.

We analysed all wet periods for all pixels in our region-of-interest, recording F_I at the end of each wet period.

References

Jong, E. J. D., Eskes, A. B., Hoogstraten, J. G. J. & Zadoks, J. C. 1987 Temperature requirements for germination, germ tube growth and appressorium formation of urediospores of Hemileia vastatrix. *Netherlands J. Plant Pathol.* **93**, 61–71. (doi:10.1007/BF01998091)

Yan, W. & Hunt, L. A. 1999 An Equation for Modelling the Temperature Response of Plants using only the Cardinal Temperatures. *Ann. Bot.* **84**, 607–614. (doi:10.1006/anbo.1999.0955)
